# Fetal growth restriction in a cohort of migrants in Germany

**DOI:** 10.1186/s12884-021-03620-z

**Published:** 2021-02-17

**Authors:** Juliane Ankert, Tanja Groten, Mathias W. Pletz, Sasmita Mishra, Gregor Seliger, Silvia M. Lobmaier, Clarissa Prazeres da Costa, Vera Seidel, Katharina von Weizsäcker, Alexandra Jablonka, Christian Dopfer, Benjamin T. Schleenvoigt

**Affiliations:** 1 Institute for Infectious Diseases and Infection Control, Jena University Hospital, Jena, Germany; 2 Department of Obstetrics, University Hospital Jena, Jena, Germany; 3 Department of Obstetrics and Gynaecology, Heidekreis Klinikum, Walsrode, Germany; 4 Polyclinic of Obstetrics and Prenatal Medicine, Halle University Hospital, Halle (Saale), Germany; 5 Department of Obstetrics and Gynecology, Klinikum rechts der Isar, Technical University of Munich, Munich, Germany; 6 Institute for Medical Microbiology, Immunology and Hygiene, Center for Global Health, Technical University of Munich, Munich, Germany; 7 Clinic for Obstetrics, Charité, University Hospital, Berlin, Germany; 8 Department of Rheumatology and Immunology, Hannover Medical School, Hannover, Germany; 9 German Center for Infection Research, Site Hannover-Brunswick, Hannover, Germany; 10 Department of Pediatric Pneumology, Allergology and Neonatology, Hannover Medical School, Hannover, Germany

**Keywords:** FGR, fetal growth restriction

## Abstract

**Background:**

Migrant women may have an increased risk of adverse birth outcomes. This study analyses the occurrence of low birth weight, preterm birth and intrauterine growth restriction / fetal growth restriction (IUGR/FGR) in pregnant migrants.

**Method:**

Cross-sectional study of 82 mother-child pairs of pregnant migrants attending medical care in Germany.

**Results:**

The Median age was 27 years, 49% of patients were of oriental-asian ethnicity and median year of migration was 2015. At least one previous pregnancy was reported in 76% of patients, in 40% the delivery mode was caesarian section. Median gestational age was 39.7 weeks. Preterm birth occurred in 6.1% of pregnancies. Median gestational age for preterm birth was 32.3 weeks. Low birth weight (< 2500 g) occurred in 6.1%. Birth weights below the 10th percentile of birth weight for gestational age were observed in 8.5% of the total cohort.

**Conclusions:**

Compared to German data no increased occurrence of low birth weight, preterm birth or IUGR/FGR was found. We note that the rate of caesarian section births was higher than in the general population for reasons yet to be identified. The authors propose stratification according to migration status for the national documentation of birth outcomes in Germany.

**Supplementary Information:**

The online version contains supplementary material available at 10.1186/s12884-021-03620-z.

## Background

The idea of intrauterine growth restriction (IUGR) was introduced in 1961 by Warkany et al. to describe the relationship between gestational age and birth weight [1]. In the more recent literature the terms intrauterine growth restriction (IUGR), fetal growth restriction (FGR) and small for gestational age (SGA) are frequently used synonymously. IUGR is increasingly being replaced by FGR [2]. It is defined as the incapability of a fetus to achieve expected growth – usually with birth weight below the 10th percentile for gestational age [3, 4]. It is expected to occur in 5–10% of all pregnancies [5] and can be compared across multi-ethnic populations [6].

Migration to Europe is a topical issue and the European Community (EU) is facing the greatest influx of refugees and migrants since the Second World War [7]. A recent study by Dopfer et al. analyzed a cohort of 2911 migrants in Germany. Within this cohort, the proportion of women of childbearing age was 18%. The authors analyzed the frequency of pregnancy among all women of fertile age and revealed a relevant pregnancy rate of 9.1 ± 0.8%. The most common country of origin of pregnant migrants was Syria (51.1%) followed by Afghanistan (21.3%) [8].

Older international studies on birth outcomes in migrant populations show heterogeneous results. A systemic review from 2010 describes that south-central Asians were more likely to have low birth weight deliveries after migration to the US and Europe, while women from Sub-Saharan Africa, Latin-America and the Caribbean were more likely to have low birth weight deliveries in Europe only [9]. The birth outcomes evaluated in this review were low birth weight (less than 2500 g) and preterm birth (gestational age < 37 weeks). A population-based study from Belgium, which examined more than 1.3 million births between 1998 and 2010, found an increased risk of perinatal mortality in all migrant groups. No low birth weight (less than 2500 g) was observed in the study population. However, a subgroup analysis showed that children born to mothers from Sub-Saharan Africa had a significantly higher risk of LBW compared to Belgians [10]. The more precise definition for IUGR/FGR/SGA, which reflects the relationship between gestational age and birth weight using birth weight percentiles, was not used in either study. A systematic review published in 2017 identified only three studies in the US and Europe, respectively, investigating the newborn risk for adverse birth outcome for this endpoint. The European studies were conducted in Scandinavia (two in Sweden and one in Denmark) [11].

To the best of our knowledge, this is the first publication from Germany investigating the frequency of adverse birth outcomes in a migrant cohort from African and Oriental Asian countries adressing FGR, defined as birth weight below the 10th percentile of the reference curves, as primary endpoint.

## Methods

We conducted a prospectively ascertained cross-sectional study using mother-child data pairs in pregnant women with a migration history from Schistosomiasis endemic countries as defined by WHO [12] attending medical care in Germany. The study was originally designed to investigate the impact of Schistosomias on birth outcome.

The Recruitment phase spanned 18 month (March 2017 to September 2018). The study was registered with the US national library of medicine (ClinicalTrials.gov Identifier: NCT03158298).

### Inclusion and exclusion criteria

Inclusion criteria: 1.) Pregnant women > 18 years, 2.) Written informed consent, 3.) Originally from endemic countries and areas for Schistosomiasis. Annotation: The corresponding countries of origin were in Africa and the Middle East.

Exclusion criteria: 1.) Fetal structural malformations, 2.) Fetal chromosomal aberrations, 3.) Multiple pregnancy. Annotation: Patients with placental pathology due to any known cause and any other medical condition affecting fetal growth were excluded.

### Assessments

A questionnaire was completed by each subject adressing demographics, age, medical history. Data concerning the primary (birth weight percentiles) and secondary pregnancy outcomes (FGR, stillbirth and premature delivery) were collected. The necessary values consisted of gestational age, weight and sex of the new born. Gestational age was determined by either ultrasound or by calculation of the last menstrual period. Blood was drawn from each woman to conduct Schistosomiasis Serology. No follow ups of newborns or mothers were performed.

### Questionnaire

Data were collected with a standardized case report form (eCRF) and pseudonymised at source. Categorical variables were: smoking, alcohol, diabetes, baby gender, ethnic origin and parity. Continuous variables were birth weight, gestational age, maternal height and weight [13]. The country of origin provided the ethnic origin more precisely. Medical conditions and concomitant medication of the mother were documented. Relevant laboratory parameters - if available from the clinical routine – were added to the dataset: Hemoglobin, Eosinophils, HIV-status and Hepatitis B and C status (Hbs-Ag, Anti-HCV).

### Ethics statement

The study was reviewed and approved by the Ethical Committee of the University Hospital Jena, Germany (approval # 4629–12/15). Follow votes were obtained for study sites in Berlin, Munich, Halle and Walsrode. All women signed an informed consent form in their native language permitting the use of their data and serum sample for scientific purposes. The study was registered with the US national library of medicine (ClinicalTrials.gov Identifier: NCT03158298).

### Statistical analysis

The statistical evaluation of the data was carried out with using SPSS Statistics Version 25. The data were first assessed for normal distribution applying a Kolmogorov-Smirnov test. For normally distributed metric data a t-test was used for independent variables and a Mann-Whitney-U-test for non-normally distributed metric data. The χ2 test was used for the analysis of nominal or ordinary data. *P*-value < 0.05 (*) were considered to be significant. Birth percentiles for height, weight and head circumference were calculated according to Voigt et al. [14]*.* In the univariate regression model, known and unknown factors that could influence the birth weight percentile were evaluated. Individual confounders with a potential influence, which had a *p*-value < 0.05 in the univariate linear regression model, were checked for multicollinearity using Kendall-Tau-B correlation analysis and included in the multiple linear regression analysis if no correlation (r < 0.5) was present. The results of the multiple linear regression analysis were considered valid if the Durbin-Watson value was between 1 and 3, Variance Inflation Factor (VIF) was < 5, the largest condition index was < 30 and *p* < 0.05.

### Schistosomiasis serology

Originally, our data collection aimed at examining the association between schistosomiasis seropositivity and adverse pregnancy outcomes. Schistosomiasis serology was negative for the whole study population. Hence, the original scientific question could not be addressed.

## Results

### Study population

82 mother-child data pairs were included. Patients were recruited at multiple sites in Germany (Jena (*n* = 35) 42.7%, Walsrode (*n* = 21) 25.6%, Halle (*n* = 13) 15.9%, Munich (*n* = 8) 9.8% and Berlin (*n* = 5) 6.1%).

The demographic characteristics of migrant women and details of the migration route are shown in Table 1. Most frequent countries of origin were Syria (35.4%) and Somalia (12.2%). Migration from African countries was observed in 40 cases (48.8%) and from Oriental Asian countries in 42 cases (51.2%).

**Table 1:** Characteristics of migrant women and their migration route

Total number of cases	*n* = 82	
Age [years; median (IQR)]	27 (11.0)	
Weight [kg; median (IQR)]	67.5 (25.0)	
BMI [kg/m^2^; median (IQR)]	24.42 (7.89)	
Year of migration to Europe; median (IQR)	2015 (2.0)	
Ethnicity [n (%)]	Oriental Asian	40 (48.8)
	African	23 (28.0)
	Caucasian	14 (17.1)
	other	5 (6.1)
Most frequent countries of origin [n (%)]	Syria	29 (35.4)
	Somalia	10 (12.2)
	Nigeria	9 (11.0)
Transportation during migration [n (%)] (multiple answers possible)	by car	16 (19.5)
	foot	20 (24.4)
	by airplane	39 (47.6)
	by boot	22 (26.8)
	train	22 (26.8)

### Medical history

The medical history included smoking in 3.7%, former smoking in 1.2% and non-smoking in 95.1%. Alcohol consumption was reported monthly or less in 4.9% and never in 95.1%. In 1.2 and 3.7% hypertension and diabetes were reported, respectively. In 8% previous anemia was evident.

### Laboratory values

Median hemoglobin was 6.98 mmol/l (IQR 1,40). HIV status was unknown/not analyzed in 53.7%, negative in 45.1% and one woman reported to be HIV positive. Hepatitis B was negative for 91.5% and unknown/not analyzed in 8.5%. Hepatitis C was negative for 17.1% and unknown/not analyzed in 82.9%.

### Previous pregnancies

75.6% of investigated women reported at least one previous pregnancy. 82% of previous pregnancies resulted in live births, 16% in abortions and 2% in stillbirths

### Current pregnancy

Gestational diabetes was reported in 8.5% and pregnancy-induced hypertension in 2.4% of patients. Pre-eclampsia occurred in 1.2%. Median weight of the mother at delivery was 78 kg (IQR 18.5). Concomitant medications during pregnancy were magnesium and methyldopa in 9.8 and 4.9%. Acetylsalicylic acid and metoprolol were not observed. Other medication was reported in 26.8%.

### Perinatal/neonatal outcomes

52% of newborns were male. The delivery mode was spontaneous in 57.3% and assisted vaginal delivery in 2.4%. Primary caesarean section and secondary caesarean section were performed in 18.3 and 22%, respectively. Median gestational age was 39.71 weeks (IQR 2.43). Median length of newborns was 51 cm (IQR 3.0). Median birth weight and head circumference were 3318 g (IQR 623) and 35 cm (IQR 2.0), respectively. Median placental weight was 500 g (IQR 105.0). Median Apgar after five and ten minutes was 10 (IQR 1 and 0). Admission to NICU was reported in 21%. No newborns died. Median umbilical cord pH was 7.29 (IQR 0.14). Preterm birth (< 37 week) occurred in 5 cases (6.1%). Median gestational age for preterm birth was 32.3 weeks (IQR 6.5). Low birth weight (< 2500 g) occurred in 5 cases (6.1%), 4 of these were preterm. Median weight for low birth weight was 1700 g (IQR 1265). *Median* percentile for weight was *35.0* (IQR 37.25). *Median* percentile for height was 38 (IQR 40.00) and median percentile for head circumference was 42.5 (IQR 41.25).

Birth weights below the 10th percentile of birth weight for gestational age were observed in 8.5% (*n* = 7) of the cohort. There was no preterm birth (< 37 week) below 10th percentile of birth weight for gestational age. Gender-specific analysis showed a difference between female (4.7%) and male (12.8%) newborns (5 vs. 2). However, the gender difference was not significant (*p* = 0.25). Thus, we observed a *fetal growth restriction (> 10%* below the 10th birth weight percentile*) for newborn boys* in our migrant population.

Univariate regression models for the birth weight percentile outcome revealed significant influence of: mother’s height, mother’s weight at delivery, transport to Europe by foot, transport to Europe by boat, number of previous pregnancies and number of previous birth (*p* **≤** 0.05 for all). Univariate regression models for birth weight revealed significant influence of: pregnancy induced hypertension, preeclampsia, previous diabetes, previous hypertension, transport to Europe by foot and number of previous birth (*p* **≤** .05 for all, data not shown).

In the multiple linear regression models on birth weight percentiles with the factors transport to Europe by foot, number of previous births and mother’s weight at delivery, a quality of 0.30 (adjusted R-square) was achieved. The results of the analysis are shown in Table 2. A significant positive effect could be demonstrated for all three factors (each *p* < .01). Transport to Europe by foot and number of previous births turned out to be equally strong (both standardized regression coefficients are 0.28 and 0.27) whereby weight at delivery with beta = 0.33 shows a comparatively higher value. The non-standardized regression coefficients B show the change in the dependent variable in one step change in the factor. If the weight of the mother at delivery increases by one unit (kilograms), the birth weight percentile increases by 0.56 units (percentiles). I.e. if the weight increases by 2 kg on delivery, the birth weight is one percentile higher. The same applies vice versa: if the weight of the mother on delivery drops by 2 kg, the birth weight is one percentile lower. If the number of previous births increases by one unit, the birth weight is 5.8 percentiles higher. The opposite applies again when the number of previous births is one unit lower. If the transport to Europe was by foot (nominal variable), the birth weight was 16.1 percentiles higher than for migrants who used another means of transport.

**Table 2:** Influence of various independent variables on birth weight percentiles - Results of the multiple linear regression models

Multiple linear Regression Depentent variable: Birth weight percentile		Transport to Europe by foot	Number of previous births	Weight at delivery
Non-standard coefficients	Regression coefficient B	16.084	5.803	0.545
	Standard Error	5.607	2.152	0.163
standard coefficient	Beta	0.278	0.271	0.333
Significance	p	0.005	0.009	0.001
95.0% Confidence intervals for B	lower limit	4.910	1.515	0.220
	upper limit	27.258	10.091	0.869
Collinearity statistics	VIF	1.018	1.097	1.079

There were seven cases of FGR (birth weight < 10th percentile) in the cohort. The numerically higher number of mothers immigrated from Syria (Syria: *n* = 4, Somalia, Nigeria and other countries respectively *n* = 1) is statistically insignificant (*p* = 0.277). Furthermore, there is no difference in occurrence after stratification by ethnicity (Oriental-Asian: n = 4 (10%), African-American: *n* = 3 (13%); *p* = 0.705).

Correlation analyses according to Kendall-Tau-B showed no correlation between birth weight and ethnicity (*p* = 0.393) and only a very weak correlation between birth weight and country of origin (r = 0.169; *p* = 0.05), which, however, is no longer evident when stratified by the respective country of origin (Nigeria, Somalia and Syria). Regression analyses showed that the different ethnicities do not differ in the degree of influence on the birth weight percentiles. Furthermore, it can be shown that none of the countries of origin has an influence on the birth weight percentiles (*p* > .7 for all).

## Discussion

Perinatal outcomes for Germany are published for 2016 and 2017 with more than 700.000 datasets per year. Unfortunately, these data were not stratified for migration background or nationality. Compared with the Germany-wide context, the data from our cohort are not noticably different regarding FGR (8.5%) and preterm birth (6.1%). Overall, preterm births in Germany occur in 6.6 and 10% of newborns are small for gestational age, i.e. below the 10th percentile of birth weight. Surprisingly, FGR is even somewhat rarer in our cohort than in a national comparison. The low rate of pregnancy-induced hypertension (2.4%) and preeclampsia (1.2%) in our cohort are in agreement with these findings. Overall, very few placental-associated pregnancy complications have been observed. However, the caesarean section rate in our cohort (40%), is remarkably higher than in the overall German data (30%). Whereas the combined endpoint of FGR and premature birth in Germany occurs in 10% but does not occur in our migrant cohort [15]. Surprisingly, admission to NICU occurred in 21% and the observed high APGAR values (10 after 5 and 10 min) do not correspond to this finding. We can only assume that there were other reasons for NICU admission after delivery during the further inpatient course that were not directly related to the birth, i.e. hypoglycemia or infection. As no follow up was conducted during the data collection the reasons for this observation remain a matter of debate.

The quality of our data is emphasized by our regression analysis, where known positive factors influencing the birth weight were confirmed (previous births and mothers weight at delivery). So far, we have no explanation for the significantly positive influence of transport to Europe by foot on birth weight percentiles observed in this study. We actually expected a reversely significant result and debated better physical training condition of the mothers due to the positive physical strain of long walking distances in the past. There is no data on this specific finding in the literature and we suggest further detailed investigation in other migrant populations.

Data on birth outcomes of the migrant population currently entering Europe are scarce; so far, no data were available for Germany. Only three European publications reflect the relationship between gestational age and birth weight to measure the frequency of adverse birth outcomes in migrant populations. Li et al. investigated more than 1 million births in Sweden from 1982 to 2006. The authors found that 4.1% of newborns born to non-Swedish mothers met the criteria for SGA, compared to 3.3% in the Swedish population [16]. In Denmark, Pedersen et al. conducted a similar analysis from 1978 to 2007 and found that migration was related to SGA rather than preterm births. The risk difference for newborns from Somalian women was 70.1% (CI 62.2 to 77.9) [17]. The third Scandinavian study compared 262 newborns of mothers from Somalia with 523 babies born in Sweden. The risk for SGA was almost three times higher in the Somalian babies (OR 2.95 CI 1.49 to 5.82). The emergency caesarean section rate was almost twice as high (OR 1.90 CI 1.16–3.10) with an approximately five-fold increased risk before the onset of labor (OR 4.96, CI 1.73–14.22) [18].

Our study was conducted in a cross sectional setting without a comparison group. We found fetal growth restriction in 8.5% of the investigated population. Compared to the results of Li et al. with data from 1982 to 2006, FGR was twice as common in our cohort (8.5% vs. 4.1%) and newborn boys were more likely to be small for gestational age (boys 12.8% vs. 4.7%). Previous studies did not differentiate by gender [16].

Taking into account that one third of our study population migrated from Syria in 2015, it can be suspected that the sequelae of war and the circumstances of forced migration entails consequences in the most vulnerable population group. This offers an explanation for our finding of a prevalence of FGR twice as high as reported by Li et al. in 2012. However, due to the small number of patients in the study subgroups, we could not confirm this assumption. On the other hand, our data are surprising compared to the German population, because they correspond to the national average both in terms of FGR and in terms of prematurity. However, the comparison of the Swedish data from 2012 with German data from 2016/2017 suggests that FGR is less common in the naïve-Swedish population than in Germany. However, the data from Germany do not include a delimitation of births of women with a migration background compared to births of naïve mothers, but are a mixture of all births in 2016/2017.

While comparing the migrant specific data from Scandinavia with our cohort the assumed increase in negative birth outcomes in migrant populations should prompt us to reconsider medical strategies for refugees in Germany, and pregnant refugees should receive particular medical attention to protect the most vulnerable group from further health damage. Especially in view of the high rate of caesarean sections in the Scandinavian study of women from Somalia (OR up to 5fold) and our secondary section rate of 22%, we should consider that we might not guide migrant women sufficiently through birth possibly due to a lack of communication which could eventually support spontaneous vaginal delivery [18]. The reasons to perform caesarean section were not analyzed in our cohort. There is also no data on this in the literature. However, a scientific comparison of our data with a more recent data set from other European countries would be appreciated. In addition, we propose stratification according to migration status for the national documentation of birth outcomes in Germany.

## Conclusions

Compared to German data no increased occurrence of low birth weight, preterm birth or IUGR was found. Of note, rate of caesarian section was higher than in the general population for reasons yet to be identified. We propose stratification according to migration status for the national documentation of birth outcomes in Germany.

## Supplementary Information

**Additional file 1.**

## Data Availability

The datasets used and/or analysed during the current study are available from the corresponding author on reasonable request.

## Abbreviations

BMI: body mass index
CI: confidence interval
eCRF: electronic case report form
EU: European Community
FGR: fetal growth restriction
HCV: hepatitis C virus
HIV: human immunodeficiency virus
IUGR: intrauterine growth restriction
IQR: interquartile range
LBW: low birth weight
NICU: Neonatal intensive care unit
OR: Odds-Ratio
SGA: small for gestational age
US: United States
VIF: Variance Inflation Factor
