# Supplementary material for: Fetal growth restriction in a cohort of migrants in Germany
Source: BMC Pregnancy Childbirth. 2021 Feb 17;21:145. doi: 10.1186/s12884-021-03620-z (PMC7890902; doi:10.1186/s12884-021-03620-z)
Supplement: Supplementary file 1 — Additional file 1. [file 12884_2021_3620_MOESM1_ESM.docx]

**
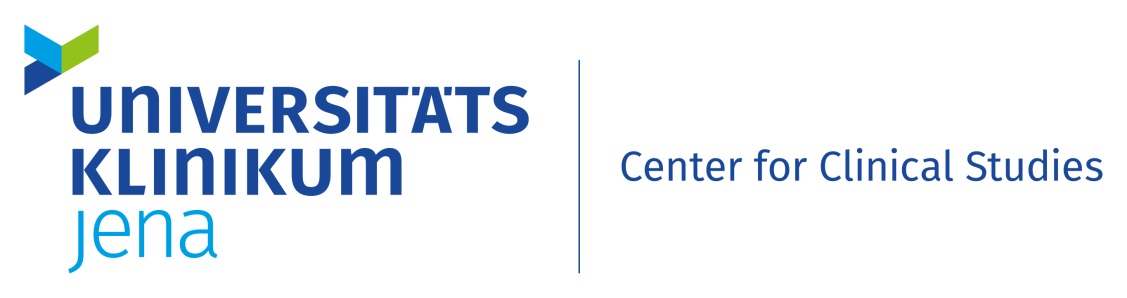
**

**CASE REPORT FORM (CRF)**

CONFIDENTIAL

Acronym: Bilharzia

ZKS-No.: ZKSJ0094

Study title: Association of Schistosomiasis seropositivity with adverse birth events in migrants from Bilharzia-endemic areas

Patient-ID: |___________|

Version: Final 02
25-Oct-2016

Principal investigator Dr. Benjamin Schleenvoigt

University Hospital Jena

Am Klinikum 1

07747 Jena

Inclusion Criteria

| Pregnant women >=18 years | 🞐 _0_ no | 🞐 _1_ yes | |
| --- | --- | --- | --- |
| Originally from endemic countries and areas for Schistosomiasis (as defined by WHO) | 🞐 _0_ no | 🞐 _1_ yes | *http://www.who.int/schistosomiasis/epidemiology/global_atlas_maps/en/* |
| Written informed consent | 🞐 _0_ no | 🞐 _1_ yes | |

Exclusion Criteria

| Fetal structural malformations | 🞐 _0_ no | 🞐 _1_ yes |
| --- | --- | --- |
| Fetal chromosomal aberrations | 🞐 _0_ no | 🞐 _1_ yes |
| Multiple pregnancy | 🞐 _0_ no | 🞐 _1_ yes |

Informed Consent

| Was the patient informed about the register and has given his written informed consent? | 🞐 _0_ no |  | |
| --- | --- | --- | --- |
|  | 🞐 _1_ yes | Date of written informed consent | \|__\|__\|.\|__\|__\|.\|__\|__\|__\|__\|  Day Month Year |

Demographic Data

| Year of birth | \|__\|__\|__\|__\| | | | |
| --- | --- | --- | --- | --- |
| Height | \|________\| cm | | | |
| Weight | \|________\| kg before pregnancy | | | |
| BMI | \|________\| kg/m^2^ calculated in OpenClinica | | | |
| Ethnicity | 🞐 _1_ Caucasian  🞐 _2_ Black  🞐 _3_ Latin-American  🞐 _4_ Oriental/Asian  🞐 _5_ Other___________________ | | | |
| Country of origin | 🞐 _1_ Algeria  🞐 _2_ Angola  🞐 _3_ Antigua  🞐 _4_ Benin  🞐 _5_ Botswana  🞐 _6_ Brazil  🞐 _7_ Burkina Faso  🞐 _8_ Burundi  🞐 _9_ Cameroon  🞐 _10_ Central Afri­can Republic  🞐 _11_ Chad  🞐 _12_ China  🞐 _13_ Congo  🞐 _14_ Cote D'Ivoire  🞐 _15_ Democratic Kampuchea  🞐 _16_ Dominican Republic  🞐 _17_ Egypt  🞐 _18_ Ethiopia | 🞐 _19_ Gabon  🞐 _20_ Gambia  🞐 _21_ Ghana  🞐 _22_ Guadeloupe  🞐 _23_ Guinea  🞐 _24_ Guinea Bissau  🞐 _25_ India  🞐 _26_ Indonesia  🞐 _27_ Iran  🞐 _28_ Iraq  🞐 _29_ Japan  🞐 _30_ Kenya  🞐 _31_ Lao People's Democratic Republic  🞐 _32_ Lebanon  🞐 _33_ Liberia  🞐 _34_ Libyan Arab Jamahiriya  🞐 _35_ Madagascar | 🞐 _36_ Malawi  🞐 _37_ Malaysia  🞐 _38_ Mali  🞐 _39_ Martinique  🞐 _40_ Mauritania  🞐 _41_ Mauritius  🞐 _42_ Montserrat  🞐 _43_ Morocco  🞐 _44_ Mozambique  🞐 _45_ Namibia  🞐 _46_ Niger  🞐 _47_ Nigeria  🞐 _48_ Oman  🞐 _49_ Philippines  🞐 _50_ Principe  🞐 _51_ Puerto Rico  🞐 _52_ Rwanda  🞐 _53_ Sao Tome  🞐 _54_ Saudi Arabia  🞐 _56_ Senegal  🞐 _57_ Sierra Leone | 🞐 _58_ Somalia  🞐 _59_ South Africa  🞐 _60_ St. Lucia  🞐 _61_ Sudan  🞐 _62_ Suriname  🞐 _63_ Swaziland  🞐 _64_ Syria  🞐 _65_ Tanzania  🞐 _66_ Thailand  🞐 _67_ Togo  🞐 _68_ Tunisia  🞐 _69_ Turkey  🞐 _70_ Uganda  🞐 _71_ Venezuela  🞐 _72_ Yemen  🞐 _73_ Zaire  🞐 _74_ Zambia  🞐 _75_ Zimbabwe  🞐 _76_ Other __________ |
| Year of migration to Europe | \|__\|__\|__\|__\| | | | |
| Smoking status | 🞐 _1_ Non-Smoker  🞐 _2_ Smoker  🞐 _3_ Ex-Smoker | | | |
| Alcohol consumption | 🞐 _0_ Never  🞐 _1_ Monthly or less  🞐 _2_ 2 to 4 times a month  🞐 _3_ 2 to 3 times a week  🞐 _4_ 4 or more times a week | | | |

Medical History

| Previous diabetes disease | 🞐 _0_ no  🞐 _1_ yes  🞐 _9_ unknown |
| --- | --- |
| Arterial hypertension | 🞐 _0_ no  🞐 _1_ yes  🞐 _9_ unknown |
| Previous Anemia (Hb < 12 g/dl resp. < 7,45 mmol/l) | 🞐 _0_ no  🞐 _1_ yes  🞐 _9_ unknown |

Bilharzia Data

| Previously known schistosomiasis | 🞐 _0_ no  🞐 _1_ yes | If yes, | Year \|__\|__\|__\|__\| 🞐 _9_ unknown (first time)  Previous therapy 🞐 _0_ no against schistosomiasis 🞐 _1_ yes  If yes, Number of therapies \|______\|  Last therapy \|__\|__\|__\|__\|  (Year) |
| --- | --- | --- | --- |
| Fresh water contact in country of origin | 🞐 _0_ no  🞐 _1_ yes  🞐 _9_ unknown | | |
| Fresh water contact on migration route to Europe | 🞐 _0_ no  🞐 _1_ yes  🞐 _9_ unknown | | |
| Means of transport to Europe (Multiple selection possible) | 🞐 _1_ by foot  🞐 _2_ car  🞐 _3_ train  🞐 _4_ boat  🞐 _5_ airplane | | |

Laboratory Values

| Hemoglobin | \|_____________\| | 🞐 _1_ mmol/l  🞐 _2_ g/dl | 🞐 _9_ not analyzed |  |
| --- | --- | --- | --- | --- |
| Eosinophils | \|_____________\| | 🞐 _1_ %  🞐 _2_ /µl | 🞐 _9_ not analyzed |  |
| HIV status | 🞐 _1_ negative  🞐 _2_ positive |  | 🞐 _9_ not analyzed |  |
| HBV status (HbsAg) | 🞐 _1_ negative  🞐 _2_ positive |  | 🞐 _9_ not analyzed |  |
| HCV status (Anti-HCV) | 🞐 _1_ negative  🞐 _2_ positive |  | 🞐 _9_ not analyzed |  |

Previous Pregnancies

| Number of pregnancies | \|_______\| | | | | |
| --- | --- | --- | --- | --- | --- |
| Number of previous births | \|_______\| | | | | |
| If >=1 previous births please fill in the table | Date of birth | Pregnancy week | Number of children | Birth weight | Delivery |
|  | \|__\|__\|__\|__\|  Year | \|_______\|  🞐 _9_ unknown | \|______\| | \|_______\| g  🞐 _9_ unknown | 🞐 _1_ spontaneous  🞐 _2_ caesarean section  🞐 _3_ prematurity (< 37. pregnancy week) |
|  | \|__\|__\|__\|__\|  Year | \|_______\|  🞐 _9_ unknown | \|______\| | \|_______\| g  🞐 _9_ unknown | 🞐 _1_ spontaneous  🞐 _2_ caesarean section  🞐 _3_ prematurity (< 37. pregnancy week) |
|  | \|__\|__\|__\|__\|  Year | \|_______\|  🞐 _9_ unknown | \|______\| | \|_______\| g  🞐 _9_ unknown | 🞐 _1_ spontaneous  🞐 _2_ caesarean section  🞐 _3_ prematurity (< 37. pregnancy week) |
|  | \|__\|__\|__\|__\|  Year | \|_______\|  🞐 _9_ unknown | \|______\| | \|_______\| g  🞐 _9_ unknown | 🞐 _1_ spontaneous  🞐 _2_ caesarean section  🞐 _3_ prematurity (< 37. pregnancy week) |
|  | \|__\|__\|__\|__\|  Year | \|_______\|  🞐 _9_ unknown | \|______\| | \|_______\| g  🞐 _9_ unknown | 🞐 _1_ spontaneous  🞐 _2_ caesarean section  🞐 _3_ prematurity (< 37. pregnancy week) |
|  | \|__\|__\|__\|__\|  Year | \|_______\|  🞐 _9_ unknown | \|______\| | \|_______\| g  🞐 _9_ unknown | 🞐 _1_ spontaneous  🞐 _2_ caesarean section  🞐 _3_ prematurity (< 37. pregnancy week) |
|  | \|__\|__\|__\|__\|  Year | \|_______\|  🞐 _9_ unknown | \|______\| | \|_______\| g  🞐 _9_ unknown | 🞐 _1_ spontaneous  🞐 _2_ caesarean section  🞐 _3_ prematurity (< 37. pregnancy week) |
| Number of abortions | \|_____\| | | | | |
| Number of stillbirths | \|_____\| | | | | |

Actual Pregnancy

| Gestational diabetes | 🞐 _0_ no  🞐 _1_ yes | |
| --- | --- | --- |
| Pregnancy-induced hypertension | 🞐 _0_ no  🞐 _1_ yes | |
| Pre-eclampsia | 🞐 _0_ no  🞐 _1_ yes | |
| Mothers weight at delivery | \|________\| kg | |
| Estimated delivery date EDD | Date \|__\|__\|.\|__\|__\|.\|__\|__\|__\|__\|  Day Month Year | Determined by 🞐 _1_ ultrasound  🞐 _2_ last menses |

Concomitant Medication (during pregnancy)

| Aspirin 100mg | 🞐 _0_ no  🞐 _1_ yes | | |
| --- | --- | --- | --- |
| Magnesium | 🞐 _0_ no  🞐 _1_ yes | | |
| Methyldopa | 🞐 _0_ no  🞐 _1_ yes | | |
| Metoprolol | 🞐 _0_ no  🞐 _1_ yes | | |
| Other | 🞐 _0_ no  🞐 _1_ yes | If yes, | ______________________ |

Newborn

| Sex | 🞐 _1_ Male  🞐 _2_ Female |
| --- | --- |
| Date of delivery | Date \|__\|__\|.\|__\|__\|.\|__\|__\|__\|__\|  Day Month Year |
| Birth | 🞐 _1_ spontaneous  🞐 _2_ vaginal surgery  🞐 _3_ primary caesarean section  🞐 _4_ secondary caesarean section |
| Gestational age | \|_____\| weeks \|_____\| days |
| Length | \|________\| cm |
| Birth weight | \|________\| g |
| Head circumference | \|________\| cm |
| Birth weight of placenta | \|________\| g |
| Apgar after 5min | \|________\| |
| Apgar after 10min | \|________\| |
| Deceased child | 🞐 _1_ alive  🞐 _2_ death |
| Umbilical cord pH | \|________\| |
| Admission to NICU | 🞐 _0_ no  🞐 _1_ yes |

Placenta Asservation

| Placenta asservation for further study purpose | 🞐 _0_ no  🞐 _1_ yes | If yes, | 🞐 _1_ in ethanol 70%  🞐 _2_ at -20°C |
| --- | --- | --- | --- |

Serology

| Result of schistosomiasis serology | 🞐 _1_ negative  🞐 _2_ positive |
| --- | --- |
